# Environmental stimuli‐responsive hydrogels in endodontics: Advances and perspectives

**DOI:** 10.1111/iej.14208

**Published:** 2025-02-06

**Authors:** He Liu, Ya Shen

**Affiliations:** ^1^ Division of Endodontics, Department of Oral Biological and Medical Sciences, Faculty of Dentistry University of British Columbia Vancouver British Columbia Canada

**Keywords:** drug delivery, endodontics, hydrogels, perspectives, stimuli‐responsive

## Abstract

Stimuli‐responsive hydrogels are smart and functional materials that respond to various environmental stimuli, including temperature, light, magnetic field, pH, redox, enzymes and glucose. This responsiveness allows for the controlled release of therapeutic agents encapsulated within the hydrogels, enhancing treatment precision, improving therapeutic outcomes and minimizing side effects. Such hydrogels show great potential in root canal disinfection, management of dental pulp inflammation and pulp regeneration, making them promising candidates for more personalized and effective endodontic treatments. This article provides an overview of the latest advancements in the design and application of stimuli‐responsive hydrogels in endodontics, emphasizing their potential to revolutionize endodontic treatments. It also addresses current challenges and explores future directions in the field, aiming to inspire and motivate researchers to further engage in or intensify their efforts within this promising area of research.

## INTRODUCTION

The dental pulp is susceptible to various physiological and pathological external stimuli, including bacterial infections, mechanical trauma, thermal changes and chemical irritants (Yu & Abbott, 2007). These factors can initiate a complex interplay with the host immune system within the pulp tissue, leading to inflammatory and immune responses (Duncan, 2022; Farges et al., 2015). When the immune system fails to regulate these responses effectively, the resulting imbalance may cause the inflammation to escalate, potentially progressing to pulp necrosis and infection (Cooper et al., 2014). Moreover, bacterial pathogens and their by‐products can spread beyond the pulp, affecting the periapical tissues and resulting in apical periodontitis—a destructive inflammatory disease characterized by a persistent inflammatory response and alveolar bone resorption in the periapical area (Wen et al., 2024). To address these pathological conditions, various therapeutic strategies such as vital pulp therapy (VPT), root canal treatment (RCT) and regenerative endodontic procedures (REPs) are employed in endodontics, focusing on effective root canal disinfection, management of dental pulp inflammation and promotion of dental pulp regeneration. For both RCT and REPs, the primary focus and challenge lie in effectively eliminating intracanal infections (Gulabivala & Ng, 2023; Liu et al., 2021). This often requires the use of intracanal medicaments to enhance infection control within the root canal. However, current intracanal medicament delivery systems are limited in their ability to achieve controlled release based on the severity of intracanal infection or periapical inflammation (Pereira et al., 2012; Ribeiro et al., 2020). If the drug concentration is too low or the duration of action is too short, it may fail to adequately control the infection. Conversely, if the concentration is too high or the duration of use is too long, it may lead to cytotoxic effects on stem cells in periapical tissues, thereby impairing the healing of periapical inflammation or the success of pulp tissue regeneration and revascularization. For VPT, the focus currently revolves around the preoperative diagnosis of the pulp condition and intraoperative assessment to determine the amount of pulp tissue to be removed or retained (Duncan, 2022). In recent years, bioactive agents have been increasingly used as adjuncts in VPT to promote the resolution of pulp inflammation and enhance reparative dentin formation (Chen et al., 2021). However, due to the lack of clinically available molecular biological tests, it remains challenging to achieve targeted and controlled drug delivery based on the degree of pulp inflammation.

Hydrogels are biocompatible and bioactive three‐dimensional networks of cross‐linked hydrophilic polymer chains (Li & Mooney, 2016). In recent years, hydrogels have garnered significant attention for their diverse applications in endodontics (Abbass et al., 2020; Huang et al., 2024; Qiu et al., 2023; Yan et al., 2021). They can serve as carriers for antimicrobial agents, enabling sustained and localized delivery within the root canal system (McIntyre et al., 2019; Yan et al., 2021; Zöller et al., 2024). They also provide a scaffold that supports cell adhesion, proliferation and differentiation (Cavalcanti et al., 2013). Additionally, hydrogels can be loaded with bioactive molecules, such as growth factors or stem cells, to promote the regeneration of dental pulp tissue, thereby creating an optimal environment for tissue healing and regeneration (Abbass et al., 2020; Huang et al., 2024; Qiu et al., 2023). Stimuli‐responsive hydrogels are smart materials that undergo physical or chemical changes in response to environmental stimuli such as pH, temperature, light, magnetic fields or enzymes and have emerged as highly attractive systems for drug delivery and tissue engineering applications (Amirthalingam et al., 2023; Conte et al., 2024; Wang, Li, et al., 2023; Wei et al., 2024; Wu et al., 2024).

This article will discuss the advantages of stimuli‐responsive hydrogels compared to traditional hydrogels, the classifications and mechanisms of stimuli‐responsive hydrogels. The advancements in therapeutic strategies utilizing stimuli‐responsive hydrogels in endodontics will also be highlighted, with a particular focus on their applications in root canal disinfection, management of dental pulp inflammation and dental pulp regeneration. Additionally, the limitations, challenges, opportunities and future directions in the development, pre‐clinical evaluation and clinical translation of these hydrogels in endodontics will be discussed. Although several recent review articles have explored the use of stimuli‐responsive hydrogels in treating oral diseases (Conte et al., 2024; Gao et al., 2024; Par et al., 2024; Wang, Li, et al., 2023; Wei et al., 2024), there remains a gap in the literature specifically addressing their application in endodontics. This article serves as a valuable resource for researchers and clinicians seeking to deepen their understanding of stimuli‐responsive hydrogels in endodontics and provides insights that could drive further advancements in the field.

## WHY DO WE NEED STIMULI‐RESPONSIVE HYDROGELS?

Stimuli‐responsive hydrogels offer significant advantages over traditional hydrogels in endodontics by providing targeted, controllable drug delivery in response to specific environmental stimuli, such as pH, temperature or enzymes. Unlike traditional hydrogels, where drug release is primarily driven by changes in gel structure (e.g. swelling, dissolution or degradation), stimuli‐responsive hydrogels can be tailored to react to specific conditions at the target site. These stimuli can trigger gel formation, drug release or degradation, allowing for precise spatiotemporal control over therapeutic interventions (Amirthalingam et al., 2023; Wei et al., 2024). This adaptability enhances their versatility in applications, improves treatment efficacy, reduces side effects and promotes better tissue integration through improved biocompatibility (Amirthalingam et al., 2023; Wei et al., 2024).

## WHAT ARE THE CLASSIFICATION AND MECHANISMS OF STIMULI‐RESPONSIVE HYDROGELS?

**FIGURE 1:**
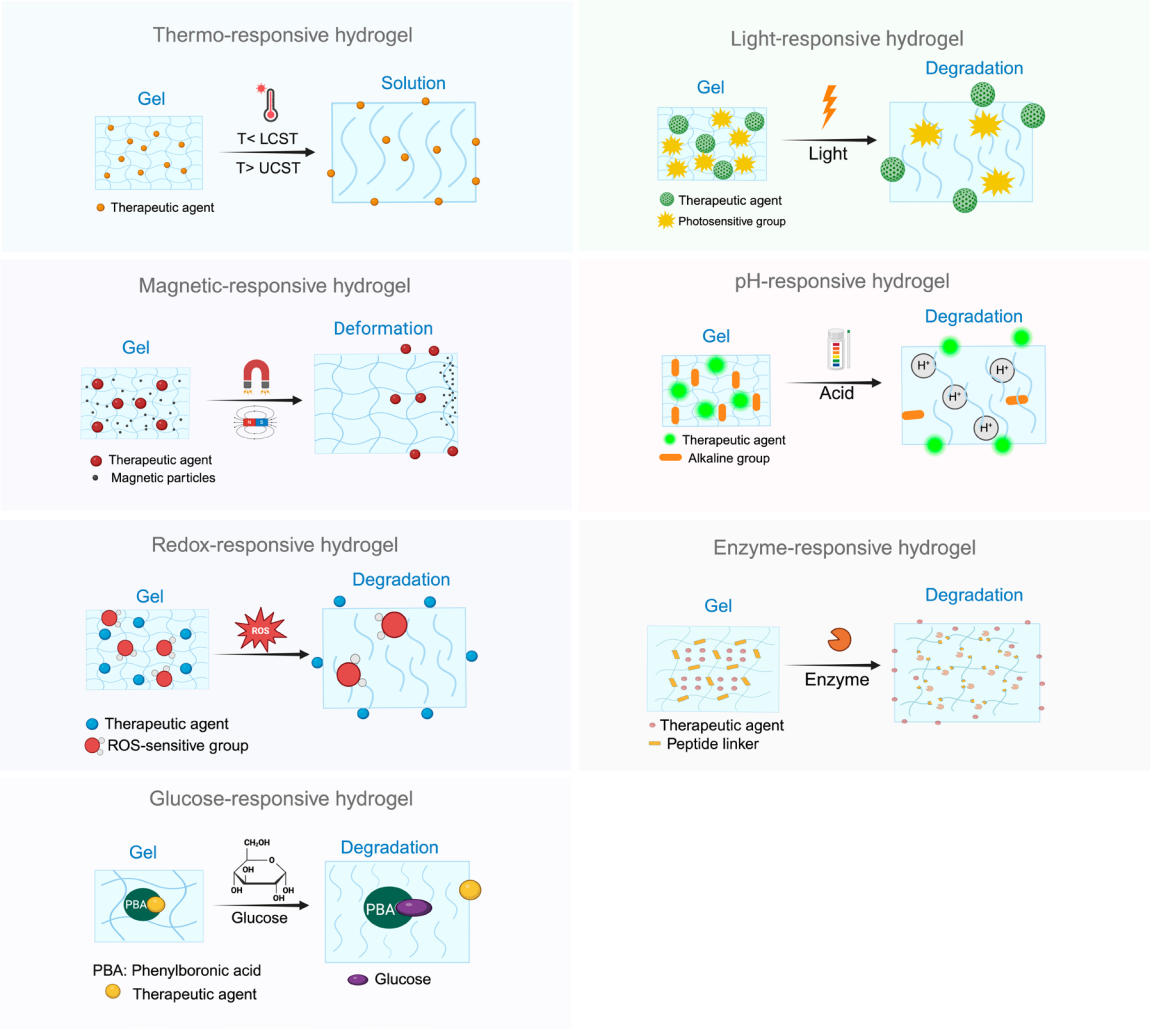
Schematic illustrations of mechanisms of stimuli‐responsive hydrogels (Created in BioRender.com).

Stimuli‐responsive hydrogels can be categorized based on the type of environmental stimuli they respond to (Amirthalingam et al., 2023; Yang et al., 2022). These include physical stimuli‐responsive hydrogels (e.g. thermo‐responsive, light‐responsive and magnetic‐responsive hydrogels), chemical stimuli‐responsive hydrogels (e.g. pH‐responsive and redox‐responsive hydrogels) and biochemical stimuli‐responsive hydrogels (e.g. enzyme‐responsive and glucose‐responsive hydrogels). It is crucial to distinguish between stimuli driving hydrogel formation (polymerization/cross‐linking/gelation) and those influencing their behaviour afterwards. This discussion focuses on stimuli‐responsive hydrogels, excluding those cross‐linked by stimuli but unresponsive to them later, as these are classified as stimuli‐cross‐linked hydrogels. Table 1 summarizes the classification and mechanisms of stimuli‐responsive hydrogels, whilst Figure 1 illustrates mechanisms of them.

**TABLE 1:** Classification and mechanism of stimuli‐responsive hydrogels.

Classification		Mechanism	Reference
Physical stimuli‐responsive hydrogels	Thermo‐responsive hydrogel	Undergo a sol–gel transition driven by hydrophobic and hydrophilic interactions at specific temperatures, affecting the hydrogel's solubility. Negative thermo‐responsive hydrogels: Characterized by a lower critical solution temperature (LCST); liquid below LCST (hydrophilic) and gel above LCST (hydrophobic) Positive thermo‐responsive hydrogels: Characterized by an upper critical solution temperature (UCST); gel below UCST and dissolve above UCST.	Amirthalingam et al., 2023; Solanki & Bhatia, 2024; Wang, Li, et al., 2023; Wei et al., 2024
	Light‐responsive hydrogel	React to light irradiation, causing structural, conformational, chemical and physical property changes. Photochemical mechanism: Light induces chemical reactions in light‐responsive groups, leading to changes in macromolecular configuration, solubility, conductivity or ion concentration, which trigger hydrogel swelling or contraction for controlled drug release. Photothermal mechanism: Light‐sensitive groups absorb energy and generate heat, raising the local temperature and inducing phase transitions similar to thermo‐responsive hydrogels, with volume changes enabling controlled drug release at the phase transition temperature.	Amirthalingam et al., 2023; Solanki & Bhatia, 2024; Wang, Li, et al., 2023; Wei et al., 2024
	Magnetic‐responsive hydrogel	Magnetic nanoparticles are embedded in a polymer matrix. Magnetic field response: Hydrogels deform, contract or expand in response to an external magnetic field, enabling controlled drug release or material movement. Localized heating: Nanoparticles generate heat in an alternating magnetic field, inducing phase changes that support controlled therapeutic applications.	Andrade et al., 2021; Fragal et al., 2022; Li et al., 2021; Liu et al., 2020; Pardo et al., 2021
Chemical stimuli‐responsive hydrogels	pH‐responsive hydrogel	Contain acidic or basic functional groups in their polymer networks, react to pH changes by swelling, shrinking or degrading, enabling targeted drug delivery in diseased tissues with adjustable degradation rates for sustained release that ceases when normal pH is restored. Polyanionic hydrogels: Swell in alkaline environments due to electrostatic repulsion and osmotic pressure. Polycationic hydrogels: Shrink in acidic environments due to electrostatic attraction.	Amirthalingam et al., 2023; Ding et al., 2022; Gupta et al., 2002; Han et al., 2023; Solanki & Bhatia, 2024; Thambi et al., 2023; Wang, Li, et al., 2023; Wei et al., 2024
	Redox‐responsive hydrogel	Hydrogels with chemical groups that react to redox changes can swell, degrade or release encapsulated substances in response to reducing or oxidizing agents. Reactive oxygen species (ROS)‐responsive hydrogels, a subtype of redox‐responsive hydrogels, incorporate ROS‐sensitive groups (e.g. thiol, phenolic hydroxyl, amide) and respond to ROS through swelling, contraction, degradation or cross‐linking changes, which affect mechanical strength and drug release.	Abed et al., 2022; Pu et al., 2024; Solanki & Bhatia, 2024; Wang, Li, et al., 2023; Wei et al., 2024
Biochemical stimuli‐responsive hydrogels	Enzyme‐responsive hydrogel	Incorporate enzyme‐cleavable motifs, using endogenous enzymes as biological triggers. Leverage enzyme‐catalysed reactions for self‐assembly and degradation. Enzyme specificity, selectivity and catalytic efficiency influence hydrogel assembly and degradation.	Amirthalingam et al., 2023; Schiffer et al., 2015; Sobczak, 2022; Solanki & Bhatia, 2024; Wang, Li, et al., 2023; Wei et al., 2024
	Glucose‐responsive hydrogel	Adapt to changes in glucose concentrations. Enable controlled drug release in diabetic tissue. High glucose levels trigger the release of therapeutic agents.	Amirthalingam et al., 2023; Chandrawati, 2016; Hu et al., 2012; Mohanty et al., 2022; Solanki & Bhatia, 2024; Wang, Li, et al., 2023

## RECENT ADVANCES IN STIMULI‐RESPONSIVE HYDROGEL STRATEGIES IN ENDODONTICS

In the past decade, the field of stimuli‐responsive hydrogels has made significant strides, establishing new standards in bio‐functional materials with notable advancements over traditional hydrogels in endodontics. These hydrogels offer unique opportunities for tailored applications in endodontics, particularly in precise drug release for root canal disinfection, management of dental pulp inflammation and pulp regeneration.

### Management of dental pulp inflammation

Inflammation of dental pulp tissue due to bacterial infection sustains elevated reactive oxygen species (ROS) levels, which further compromise the tissue's repair capacity (Dogan et al., 2023). Li, Tian, et al. (2024) developed a ROS‐responsive hydrogel by combining sodium alginate (SA) with the monomer RhB‐AC (rhodamine B‐activated carbon). SA cross‐linked with Ca^2+^ to form the first network, whilst RhB‐AC acted as a ROS‐responsive fluorescent sensor, detecting and consuming HClO/ClO^−^. The resulting SA‐RhB hydrogel effectively encapsulated dental follicle stem cell‐derived extracellular vesicles (DFSC‐sEVs), which protected dental pulp stem cells (DPSCs) from oxidative stress by regulating mitochondrial dysfunction, promoting the repair of inflamed dental pulp. The ROS‐responsive drug release was triggered by HClO/ClO^−^, showing high selectivity for this ROS and fluorescence at 580 nm indicated successful interaction. In vitro degradation tests revealed concentration‐dependent hydrogel degradation in the presence of EDTA and HClO/ClO^−^, with complete degradation in 3 days at 1000 μM. In vivo, the SA‐RhB hydrogel functioned as both a ROS scavenger and a drug carrier, clearing ROS and protecting DPSCs, offering an innovative strategy for treating dental pulp inflammation.

Accumulating research has underscored the pivotal role of MMPs in dental pulp inflammation, with MMP‐9 being particularly associated with tissue degradation in inflamed dental pulps (Agrawal et al., 2023; Mente et al., 2016; Sharma et al., 2021; Zehnder et al., 2011). Consequently, MMP‐9 may serve as a biomarker for pulpal tissue degradation, aiding in the diagnosis and monitoring of pulpitis progression or resolution (Ballal et al., 2022, 2024). This insight could facilitate the development of environmentally responsive delivery systems for therapeutic agents to manage dental pulp inflammation. Liu et al. (2024) recently developed an MMP‐9‐responsive hydrogel for VPT. The hydrogel was created by cross‐linking a custom peptide linker (CVPLS↓LYSGC), optimized for MMP‐9 sensitivity, with 4‐arm poly(ethylene glycol)‐norbornene (PEG‐NB) using thiol‐norbornene photo‐polymerization. The hydrogel incorporated the innate defence regulator peptide IDR‐1002, which was released in response to elevated MMP‐9 levels, mimicking dental pulp inflammation flare‐ups. The controlled release of IDR‐1002 enhanced its antibacterial effectiveness against multispecies oral biofilms. This MMP‐9‐responsive hydrogel offers a promising new approach for managing dental pulp inflammation.

### Root canal disinfection

The microenvironment of pathological pulp tissue or periapical lesions is acidic due to the presence of bacteria and inflammation (Nekoofar et al., 2009). Designing pH‐sensitive hydrogels that respond to this acidic environment offers a novel strategy for treating root canal infections. Chitosan hydrogels, known for their pH sensitivity due to the amine groups in chitosan, have been extensively studied for drug delivery applications (Tian & Liu, 2023). Karimi et al. (2017) developed chitosan‐based pH/thermo‐responsive hydrogels using a Schiff base reaction between chitosan and the crosslinker 2,4,6‐Tris (p‐formylphenoxy)‐1,3,5‐triazine (TRIPOD). The hydrogels were enhanced with multi‐walled carbon nanotubes, improving their mechanical properties and stability. These hydrogels exhibited pH and temperature‐dependent swelling, with significantly higher drug release in acidic environments (pH 1.5) compared to neutral (pH 6.8). This study highlights the potential of chitosan‐based hydrogels as promising intracanal delivery systems for antibacterial agents.

In apical periodontitis, MMPs play a crucial role in the host's immune and inflammatory response to root canal infections (Wahlgren et al., 2002; Wan et al., 2014). From a therapeutic perspective, MMPs could be leveraged as stimuli in the design of environmentally responsive hydrogels for root canal disinfection and the treatment of apical periodontitis. Chlorhexidine gluconate (CHX) is widely recognized for its antimicrobial efficacy and substantivity, making it a common choice as an endodontic irrigant and intracanal medication (Lessa et al., 2010; Pereira et al., 2012). However, the dose‐dependent toxicity of CHX to periapical tissues underscores the need for a delivery system that allows controlled, on‐demand release to minimize adverse effects (Ribeiro et al., 2020). In response to this challenge, Ribeiro et al. (2020) developed an injectable, MMP‐responsive, CHX‐loaded nanotube‐modified gelatin methacryloyl (GelMA) hydrogel for root canal disinfection. GelMA, which contains degradation sites for MMPs, can be further modified with aluminosilicate clay nanotubes. This functional hydrogel enables the on‐demand release of CHX encapsulated within the nanotube‐modified GelMA, triggered by elevated MMP levels associated with apical periodontitis. This study presents a controlled, on‐demand delivery strategy for therapeutic agents, utilizing enzymes such as MMPs in the periapical environment as stimuli to combat root canal infections.

### Dental pulp regeneration

Exosomes from human dental pulp stem cells (hDPSCs) have shown promise as therapeutic tools for REPs, but the rapid clearance of unconjugated exosomes limits their therapeutic efficacy (Yu et al., 2020). To address this challenge, Wang, Xing, et al. (2023) developed a thermo‐responsive hydrogel composed of hydroxypropyl chitin (HPCH) and chitin whiskers (CW) to encapsulate exosomes. In this study, exosomes were isolated from hDPSCs and directly embedded into the HPCH/CW pre‐gel to create an exosome‐loaded hydrogel (HPCH/CW/Exo). This exosome‐loaded thermos‐responsive hydrogel can be easily injected into irregular endodontic spaces and gelate in situ. In vitro cell experiments demonstrated that the delivery of exosomes significantly enhanced the hydrogel's ability to promote odontogenesis and angiogenesis. Additionally, a tooth root implantation model in nude mice confirmed the formation of new pulp‐like tissue. This study highlights the significant potential of thermos‐responsive hydrogels in REPs.

Hydrogels containing chitosan have been extensively used as scaffolds for DPSCs in REPs due to their ability to enhance the growth, attachment and odontoblastic differentiation of DPSCs (Aguilar et al., 2019). Chitosan–gelatin composite scaffolds have garnered significant attention in tissue engineering and regeneration because they closely resemble the ECM of natural tissues, and they offer excellent biocompatibility and biodegradability (Bakopoulou et al., 2019; Ducret et al., 2019; Vagropoulou et al., 2021). Chen et al. (2016) developed and evaluated an injectable thermo‐sensitive hydrogel composed of chitosan, β‐glycerophosphate and hydroxyapatite. The chitosan/β‐glycerophosphate hydrogel and chitosan/β‐glycerophosphate/hydroxyapatite hydrogel were prepared using the sol–gel method. The results demonstrated that this hydrogel exhibited excellent cellular compatibility and effectively promoted the osteogenic differentiation of dental pulp stem cells. Samiei et al. (2023) designed injectable thermo‐responsive chitosan/gelatin hydrogels to deliver DPSCs for REPs. In their study, Poly(N‐isopropylacrylamide) (PNIPAAm) was employed to create hydrogels that undergo sol–gel transitions around 33°C. Acid‐terminated PNIPAAm was synthesized and grafted onto chitosan (PNIPAAm‐g‐chitosan) via an amide condensation reaction, and the resulting copolymer was mixed with various ratios of gelatin solution and cross‐linked using genipin to form PNIPAAm‐g‐chitosan/gelatin hybrid hydrogels. These hydrogels, which combine thermo‐responsiveness with biocompatibility and biodegradability, promote the osteogenic differentiation of DPSCs and enhance the expression of osteogenic genes and calcium deposition. Therefore, these thermo‐responsive hydrogels can create osteogenic microenvironments for DPSCs in REPs.

## STIMULI‐RESPONSIVE HYDROGEL STRATEGIES IN ENDODONTICS: LIMITATIONS AND CHALLENGES

### What are the limitations of using a single stimulus?

In the development of stimuli‐responsive hydrogels for endodontics, each type of stimulus offers specific advantages but may not be suitable for all therapeutic contexts. For instance, light stimulus might not effectively reach the apical part of the root canal system, potentially compromising the hydrogel's responsiveness—such as curing, swelling or degradation—and thus affecting its functionality. To address these limitations, researchers have developed multi‐stimuli‐responsive hydrogels that can respond to multiple triggers, enhancing their adaptability and effectiveness. For example, dual light/temperature‐responsive hydrogels may offer improved spatiotemporal control in the apical region. Integrating various stimuli‐responsive elements into these hydrogels further enhances their sensitivity, adaptability and multifunctionality, making them promising candidates for advanced endodontic therapies and broader medical applications.

### What challenges are involved in designing stimuli‐responsive hydrogels with respect to specificity, sensitivity and microenvironment variability?

Stimuli‐responsive hydrogels must demonstrate high specificity and sensitivity to the external stimuli they target. For instance, in a stimuli‐responsive hydrogel designed to target an enzyme in the inflammatory microenvironment of dental pulp, such as MMP‐9, the selected peptide linker must exhibit high specificity and sensitivity to MMP‐9. This specificity and sensitivity must be thoroughly evaluated through laboratory and animal studies. Poor specificity could result in other stimuli triggering hydrogel degradation and the release of therapeutic agents, leading to misguided release, potentially compromising biocompatibility and causing side effects. If sensitivity is insufficient, the hydrogel may not release an adequate amount of therapeutic agents. Apart from the challenges in the material design of stimuli‐responsive hydrogels, variability in the microenvironments within diseased tissues amongst patients can significantly impact the performance of these hydrogels, particularly when they are designed to respond to endogenous stimuli under pathological conditions (Amirthalingam et al., 2023; Kirschner & Anseth, 2013). However, there is a lack of robust physiological data during disease progression or the natural healing cascade of injured sites, and significant patient‐to‐patient variation exists in the concentration and presence of biological molecules (Kirschner & Anseth, 2013). For instance, the expression of inflammatory mediators such as interleukin‐8 (IL‐8), MMP‐9 and tumour necrosis factor‐α (TNF‐α) increases in inflamed dental pulp (Zanini et al., 2017). Recent preliminary studies have shown that elevated levels of MMP‐9 in pulpal blood correlate with the severity of pulpitis. However, the exact concentration of MMP‐9 across different stages of pulpitis, as well as its cut‐off value, remains unclear. This uncertainty poses significant challenges in designing MMP‐9‐responsive hydrogels for VPT in terms of their sensitivity and effectiveness (Liu et al., 2024).

It is pivotal to advance basic research and clinical studies to elucidate the mechanisms and microenvironmental changes related to the physiology and pathology of dental pulp and periapical tissues, in parallel with the design, evaluation and application of advanced functional materials such as stimuli‐responsive hydrogels in endodontics (Zöller et al., 2024). When designing stimuli‐responsive hydrogels for dental pulp regeneration, the degradation profile of the hydrogel must match the tissue regeneration profile. It is essential to consider factors influencing the degradation of the stimuli‐responsive hydrogel itself (such as chemical composition, type of cross‐linking, material morphology and nanofillers or additives), factors related to the dental pulp tissue and the inflammatory microenvironment (such as the regeneration speed of pulp tissue, external stimuli and environmental conditions) and the interaction between the stimuli‐responsive hydrogel and the dental pulp tissue (such as the hydrogel's biocompatibility, mechanical properties, porous structure and the loaded cells and bioactive factors).

### What are the clinical challenges in translating stimuli‐responsive hydrogels into endodontic practice?

Although recent studies have reported promising results regarding the development and evaluation of stimuli‐responsive hydrogels in endodontics, these hydrogels are expected to face significant clinical hurdles. First, the cost of development: extensive cell experiments and animal model studies are still required to gather sufficient evidence on biocompatibility, biosafety and efficacy before clinical testing can proceed. Second, the regulatory cost: the pathway to clinical application is filled with regulatory challenges, especially for materials incorporating active biological components, which require more rigorous clinical trials (Duncan et al., 2023). Third, the production and market feasibility: the synthesis processes for stimuli‐responsive hydrogels are complex, time‐consuming and expensive, making commercial mass production difficult, which in turn hampers the large‐scale application of these hydrogels (Wei et al., 2024).

Several strategies can be implemented to address the clinical translation challenges of stimuli‐responsive hydrogels in endodontics. First, optimize development costs using more efficient synthesis and testing methods, such as advanced computer simulations or artificial intelligence (AI) technologies to predict hydrogel performance, reducing the need for extensive lab and animal testing (Li, Song, et al., 2024). Second, streamline the regulatory process by collaborating closely with regulatory agencies and exploring the repurposing of already approved materials or drugs to bypass early‐stage clinical trials. Third, improve production efficiency by adopting scalable automation technologies and sustainable raw materials, simplifying the synthesis process and making large‐scale manufacturing more feasible. Finally, enhance market adoption by partnering with industry and investors, highlighting the clinical benefits and market potential of hydrogels to attract funding and investment.

## FUTURE OPPORTUNITIES AND DIRECTIONS

Despite their promising potential, research on the development and pre‐clinical evaluation of various stimuli‐responsive hydrogels in endodontics is still in its early stages. Researchers should deepen their understanding of these materials in the context of endodontics and intensify their efforts in exploring this exciting and promising area of research. Stimuli‐responsive hydrogels currently being developed for endodontic applications leverage environmental stimuli like pH, enzymes and ROS. Research indicates that diabetes can impair dental pulp metabolism and increase ROS levels, leading to heightened inflammation and tissue degeneration (Leite et al., 2008; Pimenta et al., 2024). Glucose‐responsive hydrogels have been successfully utilized for controlled drug release in diabetic tissues and for enhancing diabetic wound healing (Chen et al., 2023; Wang et al., 2024). These designs could be adapted or further refined to create glucose‐responsive antioxidant hydrogels aimed at effectively reducing ROS levels in diabetic pulp tissue, thereby mitigating inflammation. Magnetic nanoparticles‐incorporated root canal sealers have demonstrated enhanced penetration in root canals both in vitro and in vivo, improving sealing efficacy and reducing the risk of bacterial reinfection (Guo et al., 2022). Magnetic‐responsive hydrogels hold significant potential in endodontics due to their ability to respond to external magnetic fields. These hydrogels can be loaded with antibacterial or anti‐inflammatory agents and precisely directed to targeted areas within the root canal system. Additionally, they allow for remote activation via magnetic fields, enabling controlled, on‐demand release of therapeutic agents, making them particularly beneficial for managing postoperative inflammation and recurrent infections.

Innovative fabrication techniques, such as 3D and 4D bioprinting, offer exciting opportunities for advancing the design of stimuli‐responsive hydrogels in endodontics. Specifically, 3D bioprinting allows for the precise layer‐by‐layer deposition of bio‐inks, enabling the creation of customized hydrogel structures embedded with living cells, growth factors and biomaterials (Faber et al., 2023). This technology holds great potential for producing tailored tissues that closely mimic the natural architecture of dental tissues, paving the way for future applications in tissue engineering and regenerative endodontics. On the contrary, 4D bioprinting adds a dynamic element by incorporating the dimension of time, allowing printed hydrogels to change shape or properties in response to environmental stimuli like temperature, pH or mechanical forces (Faber et al., 2023). This adaptability makes 4D‐printed hydrogels particularly suitable for endodontics, where the material will respond to the unique microenvironment within pulp tissue, root canal space or periapical lesion, such as inflammation or infection. However, the clinical translation of 4D bioprinting depends on selecting suitable stimuli‐responsive biomaterials, applying safe and effective external stimuli, employing rational design strategies for shape morphing and utilizing efficient additive manufacturing techniques (Yarali et al., 2024). Advancing multi‐material printing methods to combine different bio‐inks will further enhance the complexity and functionality of stimuli‐responsive hydrogels (Lai et al., 2024; Yarali et al., 2024). Moreover, integrating patient‐specific data into 4D bioprinting, such as dental imaging scans, will enable the creation of personalized hydrogel scaffolds tailored to individual clinical conditions in endodontics (Lai et al., 2024).

## THE POTENTIAL OF STIMULI‐RESPONSIVE HYDROGELS TO REVOLUTIONIZE THE PARADIGM OF ENDODONTIC TREATMENT MODALITIES

The past 10 years have witnessed a paradigm shift in endodontic treatment, driven by a deeper understanding of pulpal biology, the development of new materials and improved treatment strategies (Duncan, 2022). Clinical research has highlighted the role of VPT in managing teeth with signs and symptoms indicative of irreversible pulpitis (Duncan, 2022). However, VPT procedures typically involve excising inflamed or infected pulp tissue and sealing the remaining pulp with hydraulic calcium silicate cements, which may not fully resolve inflammation for effective pulp healing (Ricucci et al., 2020). Bioactive agents have been used as adjuncts in VPT to promote pulp inflammation resolution and enhance reparative dentin formation (Chen et al., 2021). Yet, without a clinical test to accurately diagnose and measure pulpal inflammation, determining the optimal condition for applying these bioactive agents remains challenging. Recently, stimuli‐responsive hydrogels triggered by ROS or MMP‐9 in the pulp tissue have offered innovative strategies in VPT for managing dental pulp inflammation (Li, Tian, et al., 2024; Liu et al., 2024). Although the clinical application of these hydrogels remains distant, translating the findings of this perspective into real‐world practice could lead to significant clinical outcomes. Stimuli‐responsive hydrogels have the potential to enhance targeted treatment in endodontics by enabling controlled drug release in response to inflammatory markers like ROS or MMP‐9. This innovation could improve the management of pulp inflammation and infection, offering more personalized and effective therapies. However, to successfully integrate these hydrogels into clinical practice, further in vivo studies are necessary to confirm their efficacy and safety. Additionally, the development of clinical diagnostic tools to accurately assess pulpal inflammation will be crucial for optimizing the application of these bioactive materials. By addressing these challenges, clinical outcomes could be enhanced, leading to more effective treatment strategies in endodontics.

## CONCLUSIONS

Recent advances in stimuli‐responsive hydrogels hold great potential for tailored applications in endodontics, particularly in precise drug release for root canal disinfection, management of dental pulp inflammation and pulp regeneration. To fully harness their potential, several key areas require attention: Future efforts should focus on creating multi‐stimuli‐responsive hydrogels and specialized hydrogels (such as glucose‐responsive hydrogels for diabetic patients); advancing basic and clinical research is essential to understand the physiological and pathological changes in dental pulp and periapical tissues; successfully translating these hydrogels to clinical use requires optimizing development processes, streamlining regulatory pathways and improving production efficiency; and further in vivo studies are needed to verify the safety and efficacy of these hydrogels in clinical settings.

## AUTHOR CONTRIBUTIONS

He Liu and Ya Shen conceived the idea. He Liu wrote and prepared the original draft. He Liu and Ya Shen revised and edited the manuscript. All authors have reviewed and approved the final manuscript.

## FUNDING INFORMATION

The work was not supported by funding.

## CONFLICT OF INTEREST STATEMENT

The authors declare no conflict of interest.

## ETHICAL APPROVAL

This work did not involve human or animal subjects.

## Data Availability

Data sharing is not applicable to this article as no datasets were generated or analysed during the current work.
